# Association between Obesity and Anemia in a Nationally Representative Sample of South Korean Adolescents: A Cross-Sectional Study

**DOI:** 10.3390/healthcare10061055

**Published:** 2022-06-06

**Authors:** Jaehoon Jeong, Younghoon Cho, In-Young Cho, Joonho Ahn

**Affiliations:** 1 Department of Medical Care, Jeju Correctional Institution, Jeju 63147, Korea; wjwogns@hanmail.net; 2 Yeoncheon-gun Public Health and Medical Center, Jeongok-eup, Yeoncheon-gun 11017, Korea; tccyh@hanmail.net; 3 Kangbuk Samsung Hospital, Sungkyunkwan University School of Medicine, Seoul 03181, Korea; 4 Department of Occupational and Environmental Medicine, Seoul St. Mary’s Hospital, College of Medicine, The Catholic University of Korea, 222, Banpo-daero, Seocho-gu, Seoul 06591, Korea

**Keywords:** obesity, pediatric obesity, anemia, adolescents

## Abstract

Anemia is associated with physical, cognitive, and developmental problems. Given that there are limited studies on anemia prevalence among obese Asian adolescents and that past evidence is controversial, this study investigated the relationship between obesity and anemia in a nationally representative sample of South Korean adolescents. Data were obtained from the 2007–2019 Korea National Health and Nutrition Examination Survey. Overall, 10,231 subjects were included in the analysis. Multiple logistic regression was performed to determine the relationship between obesity and anemia. Compared with the non-obesity group, the adjusted odds ratio (OR; 95% confidence interval [CI]) of anemia was 1.00 (0.66–1.50) in the obesity group. However, in the early adolescent subgroup of 10–13 years (adjusted OR, 2.88; 95% CI, 1.20–6.95), the risk of anemia was significantly higher in the obese group than in the non-obese group. Obesity was associated with an increased risk of anemia in early adolescents. Obese adolescents aged 10–13 require special care, especially through regular examination and management for anemia.

## 1. Introduction

Childhood obesity has emerged as one of the most important public health problems in Korea and other countries worldwide [1,2]. The increasing prevalence of childhood obesity has led to the emergence of multiple serious obesity-related comorbidities that not only pose a threat to the health of those affected but also place a large burden on the health care system [3,4]. Childhood and adolescent obesity can progress to adult diseases, early onset of metabolic syndrome, joint disease, and mental problems such as feelings of inferiority and depression [5].

Anemia is a condition characterized by a decreased number of red blood cells (RBCs), resulting in insufficient oxygen-carrying capacity of the RBCs to meet the physiological needs of the body [6]. Anemia has been associated with decreased exercise capacity, impaired cognitive function, developmental delay, and behavioral disturbances [7]. Therefore, anemia is an important health problem among adolescents, who undergo rapid physical and mental changes, as it can affect normal growth and development [8].

Some studies have reported that anemia is more prevalent in obese individuals, suggesting a link between obesity and anemia [9,10,11]. Studies have also shown that obese adults and adolescents have a higher risk of anemia [12,13,14]. This is most likely due to insufficient dietary iron intake or inflammation of fat cells, but the exact cause remains unclear [15,16]. However, there are also studies reporting that anemia is less common in obese adolescents [17,18], which remains controversial. Particularly, not many studies have assessed Asian populations, and some studies have assessed only a small group of adolescents in South Korea [19,20].

The purpose of this study was to investigate the relationship between obesity and anemia in obese children and adolescents based on data from the Korea National Health and Nutrition Examination Survey (KNHANES) [21], which includes representative data of the Korean population.

## 2. Materials and Methods

### 2.1. Data Collection and Study Participants

This was a cross-sectional study using data from the 2007–2019 KNHANES [21]. The KNHANES is a cross-sectional, nationally representative, population-based survey conducted by the Korea Centers for Disease Control and Prevention, examining approximately 10,000 people each year. The health interviews and health examinations were conducted by trained medical staff and interviewers in mobile examination centers.

**Figure 1:**
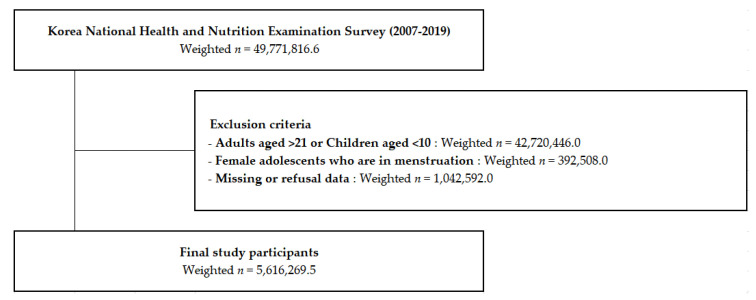
Schematic diagram of the participant selection process.

The source population included 105,732 baseline respondents (weighted *n* = 49,771,816.6) from the 2007–2019 KNHANES (Figure 1). After excluding participants who were more than 21 years old and less than 10 years old (weighted *n* = 42,720,446.0), who had menstruation at the time of examination (weighted *n* = 392,508.0), and who had missing variables (weighted *n* = 1,042,592.0), the final sample analyzed had 10,231 participants (weighted *n* = 1,042,592.0).

### 2.2. Obesity

The KNHANES provided information on the participants’ height and weight, which was measured by trained medical staff. For those aged 10 years and older, their height was measured while standing, and their weight was measured while wearing a gown for the health examination. The body mass index (BMI) is calculated based on the height and weight, and obesity is defined as a body mass index (BMI) of ≥25 kg/m^2^ for adults [22]. However, in the case of children and adolescents, there is no specific BMI cutoff for obesity; the BMI percentile based on the child or adolescent’s age is used to evaluate the presence or absence of obesity. Therefore, we attempted to evaluate the prevalence of obesity using the percentile of the BMI, which was based on the 2017 Child Growth and Development Table [23]. Obesity was defined when the BMI percentile was ≥95th according to each participant’s age.

### 2.3. Anemia

Anemia is defined as a hemoglobin concentration of less than the fifth percentile for age. Normal hemoglobin levels vary by age. Hence, a patient’s hemoglobin level must be compared with age-based norms to diagnose anemia. According to the World Health Organization (WHO) guidelines [24], anemia was defined as a hemoglobin concentration of less than 11.5 g/dL for individuals aged 10–11 years and a hemoglobin concentration of <12 g/dL for those aged 12–14 years. For those aged ≥15 years, the cutoff is set differently for male and female, and follows the criteria for adults. Anemia was defined as a hemoglobin concentration of <12 g/dL for a nonpregnant woman, that of <11 g/dL for a pregnant woman, and that of <13 g/dL for a man.

### 2.4. Other Variables

The survey assessed sociodemographic and health-related characteristics, including sex, age, household income, and menstruation. According to the Nelson Textbook, adolescence is divided into three stages, from the age of 10 years to the age of 21 years [25]: early adolescence (10–13 years old), middle adolescence (14–17 years old), and late adolescence (18–21 years old). Therefore, adolescence was analyzed according to these three stages: early (10–13 years), middle (14–17 years), and late (18–21 years). Household income was classified into four categories based on the median value: low, mid-low, high-middle, and high. Menarche was divided into two responses: before menarche and after menarche.

### 2.5. Statistical Analysis

In the KNHANES, all statistical data were calculated using sample weights. The sample weights were constructed for sample participants to represent the Korean population by accounting for the complex survey design and survey nonresponse.

To approximate the Korean population, we calculated the weighted values using a cluster sampling weight that was estimated from the KNHANES’s complex sampling design. Univariate and multivariate logistic regression analyses were conducted to adjust for confounding factors and evaluate the relationship between obesity and anemia. Confounding factors included age group, sex, household income, and menarche in girls. Statistical analyses were conducted using SAS software (version 9.4, SAS Institute, Cary, NC, USA).

## 3. Results

### 3.1. Characteristics of the Study Participants

The characteristics of the participants are shown in Table 1. Participants who were female, had older age, and had experienced menarche were likely to have anemia. However, there was no significant difference in household income and obesity between the non-anemia and anemia groups.

### 3.2. Association between Obesity and Anemia in Adolescents

The adjusted odds ratios (ORs) (95% confidence intervals [CIs]) of anemia, with the non-obesity group as the reference, were 1.00 (95% CI, 0.66–1.50; Table 2). For all adolescents, the relationship between obesity and anemia did not show any statistically significant result.

When stratified by age group, there was no significant increase in risk in obese adolescents aged 14–17 years (adjusted OR, 0.63; 95% CI, 0.31–1.29) and 18–21 years old (adjusted OR, 1.10, 95% CI, 0.64–1.91). However, in the age group of 10–13 years (adjusted OR, 2.88, 95% CI, 1.20–6.95), the risk of anemia was significantly higher in the obese group than in the non-obese group. When stratified according to sex, there was no significant increase in the risk of anemia in both males (adjusted OR, 1.01; 95% CI, 0.26–3.90) and females (adjusted OR, 1.01; 95% CI, 0.66–1.54).

The crude and adjusted ORs (95% CIs) of anemia in female adolescents, with the non-obesity group as the reference, were 1.09 (0.72–1.66) and 0.96 (0.63–1.48), respectively. After stratifying female adolescents into three age groups, the OR was 3.05 (1.25–7.43) in the early adolescent age group. Although the results were analyzed by adjusting for menarche in female adolescents, the results were statistically significant.

## 4. Discussion

In the present study, the relationship between obesity and anemia did not show any statistically significant results for all adolescents, but the risk of anemia was significantly higher in obese adolescents who were in early adolescence (10–13 years).

The relationship between obesity and anemia is controversial. Many studies have reported controversial results: some studies reported that obesity increases the risk of anemia [9,11,26], whereas others reported that obesity lowers the risk of anemia [19,27]. The association between obesity and iron deficiency was first revealed by Wenzel et al. in 1962 [28]. Since then, many studies and research results have reported that the prevalence of anemia increases with obesity [9,11,26]. The results in children were the same as those in adults [10,29,30,31,32]. On the contrary, other studies reported that iron deficiency is decreased in obese children compared to non-obese children because of a higher nutritional intake. In a study conducted on middle school and female middle school students in Korea, the risk of iron deficiency was higher in non-obese children [19,20]. In a study conducted in India, underweight children who had a lower intake of iron-containing animal foods had a higher risk of anemia than overweight or obese children [27].

The WHO report on anemia defines growing adolescents and reproductive-age women as high-risk groups for anemia [24]. Some studies reported that the prevalence of anemia is higher in adolescents who are obese; it is reported that the anemia occurs because the iron intake is not maintained as the iron requirement increases in adolescents who are undergoing a second rapid growth period. In males, secondary sexual characteristics begin in early adolescence, followed by growth spurt in middle adolescence [25]. Girls begin growth spurts in early adolescence and experience a peak growth velocity in middle adolescence [25]. Taken together, we can assume that the age group of 10–13 years is the key period in which growth occurs most rapidly among adolescents and the period in which metabolism and body changes occur most actively. In this period, if there is a nutritional imbalance owing to the lack of a balanced diet; then, severe nutritional deficiencies can occur because the need for essential nutrients increases rapidly [33,34,35]. Obesity can be thought of as a state of overnutrition, but obese adolescents with malnutrition are more likely to be more vulnerable to the deficiency of essential nutrients [36,37,38]. Therefore, adolescents experiencing rapid growth may be more vulnerable to anemia if they are obese. Obesity can also be seen as a state of chronic inflammation [39,40]. Cytokines and inflammatory substances secreted in this state of chronic inflammation can cause iron deficiency [41]. Impaired functional iron status is mainly linked to adipose tissue inflammation and increased expression of the systemic iron regulatory protein hepcidin [26,42]. A study of adolescents in Indonesia and Thailand showed that obese adolescents had a higher risk of anemia, which was explained in the context of inflammation [16,43]. In addition to this mechanism, the increased risk of anemia in obese adolescents can be explained by unhealthy eating habits [44]. Adolescents are more likely to become obese due to overeating, but obesity does not mean balanced nutrition [45]. According to the results of a recent survey on the dietary habits of Korean adolescents, there have been increasing trends of obesity in adolescents who skip breakfast, and consume excess fast food and sugary drinks [46]. A study conducted in Serbia found that children who consumed excess fast food had an increased risk of anemia [47].

Anemia in female adolescents can be considered due to menstruation [48]. However, women who were menstruating at the time of the blood test were excluded, and the relationship between obesity and anemia was still significant even after adjusting for menarche in the female adolescent group aged 10–13 years. This suggests that our study results are unlikely to be affected simply by menstruation or menarche. We also tried to compare the differences by sex, but the results could not be calculated for male adolescents aged 10–13 years owing to an insufficient sample size. The sex differences vary widely between studies, and this is likely due to dietary habits and many cultural factors [30,31,32]. Therefore, it is difficult to draw a specific conclusion because various results are presented according to the population characteristics, such as the culture, region, and race. Further research including different population groups and sufficient sample size to perform analysis by sex is needed in the future.

To the best of our knowledge, this is the first study to demonstrate a significant association between obesity and anemia in Korean obese adolescents who are in early adolescence using a large-scale representative sample. Although there have been studies that analyzed the relationship between BMI and anemia in adults, there have been no studies on this topic among Korean adolescents. However, this study also has limitations, the first being its cross-sectional design, which was not sufficient to confirm the causality between obesity and anemia. Therefore, a longitudinal study is required in the future. In addition, because the analysis was performed including variables available in the KNHANES, all factors that may affect anemia could not be included, such as lifestyle behaviors among children and adolescents. Moreover, because the results were analyzed only for the presence or absence of anemia, the exact cause and types of anemia remained unknown, warranting investigation in future studies.

## 5. Conclusions

Anemia is usually a curable disease, and special attention should be paid to adolescents aged 10–13 years. Obese individuals may be vulnerable to anemia, necessitating the importance of regular examinations and management in this population. Clinicians who are treating or managing the health of an obese adolescent in early adolescence should be aware that anemia may be present.

## Figures and Tables

**Table 1:** Weighted prevalence of anemia according to the characteristics of the study participants.

		Anemia			Non-Anemia		*p*-Value ^d^
	*n* ^a^	E ^b^	% ^c^	*n* ^a^	E ^b^	% ^b^	
Total	185,731	11,685	3.307	5,430,539	35,528	96.693	
Gender							<0.001
Male	12,697	2845	0.401	3,171,566	38,876	99.601	
Female	173,033	11,353	7.115	2,258,973	33,935	92.890	
Age (year)							<0.001
10–13	13,744	2475	0.809	1,686,183	25,283	99.191	
14–17	75,907	7115	3.714	1,967,812	33,158	96.286	
18–21	96,080	9038	5.131	1,776,544	37,965	94.869	
Obesity							0.6396
Obesity	22,881	4180	3.058	725,419	23,227	96.942	
Non-obesity	162,850	10,945	3.345	4,705,120	37,283	96.655	
Household income							0.1490
1 quartile	31,516	5086	4.228	713,968	23,989	95.772	
2 quartile	48,932	5785	3.298	1,434,877	31,145	96.702	
3 quartile	59,227	6651	3.507	1,629,478	30,090	96.493	
4 quartile	46,055	5956	2.712	1,652,216	30,276	97.288	
Menarche (Girls)							<0.001
Before menarche	2001	1064	0.455	437,305	14,101	99.545	
After menarche	171,033	11,145	8.583	1,821,667	25,946	91.417	

^a^ Weighted sample size. ^b^ Standard error. ^c^ Weighted percentage. ^d^ Weighted *p* value.

**Table 2:** Odds ratios and 95% confidence intervals for the relationship between obesity and anemia using a logistic regression model.

	Model 1 ^a^ OR (95% CI)	Model 2 ^b^ OR (95% CI)	Model 3 ^c^ OR (95% CI)
Total	0.91 (0.62–1.35)	1.00 (0.66–1.50)	N/A ^d^
Gender			
Male	1.08 (0.28–4.11)	1.01 (0.26–3.90)	N/A ^d^
Female	1.09 (0.72–1.66)	1.01(0.66–1.54)	0.96 (0.63–1.48)
Age group			
10–13 (early)	**2.48 (1.10–5.55)**	**2.88 (1.20–6.95)**	N/A ^d^
14–17 (middle)	0.67 (0.34–1.31)	0.63 (0.31–1.29)	N/A ^d^
18–21 (late)	0.77 (0.45–1.30)	1.10 (0.64–1.91)	N/A ^d^
Male group			
10–13 (early)	N/A ^e^	N/A ^e^	N/A ^d^
14–17 (middle)	1.66 (0.29–9.56)	1.68 (0.29–9.70)	N/A ^d^
18–21 (late)	0.85 (0.10–7.39)	0.86 (0.10–7.78)	N/A ^d^
Female group			
10–13 (early)	**3.66 (1.59–8.41)**	**3.77 (1.54–9.26)**	**3.05 (1.25–7.43)**
14–17 (middle)	0.58 (0.28–1.23)	0.56 (0.26–1.19)	0.57 (0.27–1.22)
18–21 (late)	1.13 (0.65–1.98)	1.12 (0.64–1.97)	1.14 (0.65–2.00)

OR, odds ratio; CI, confidence interval. ^a^ Model 1: Crude ratio ^b^ Model 2: Adjusted for sociodemographic factors (sex, age, and household income). ^c^ Model 3: Model 2 plus adjusted for menarche. ^d^ N/A: not applicable for both genders. Adjustment for menarche applies only for females. ^e^ N/A: not applicable due to the small sample size. Bold format indicates statistically significant index.

## Data Availability

Not applicable.
